# Supplementary material for: Diagnostic Application of Volatile Organic Compounds as Potential Biomarkers for Detecting Digestive Neoplasia: A Systematic Review
Source: Diagnostics (Basel). 2021 Dec 9;11(12):2317. doi: 10.3390/diagnostics11122317 (PMC8700395; doi:10.3390/diagnostics11122317)
Supplement: Supplementary file 1 [file diagnostics-11-02317-s001.zip › diagnostics-1468598-supplementary.pdf]

Supplementary File 1. Literature Search strategy

Pubmed/ MEDLINE

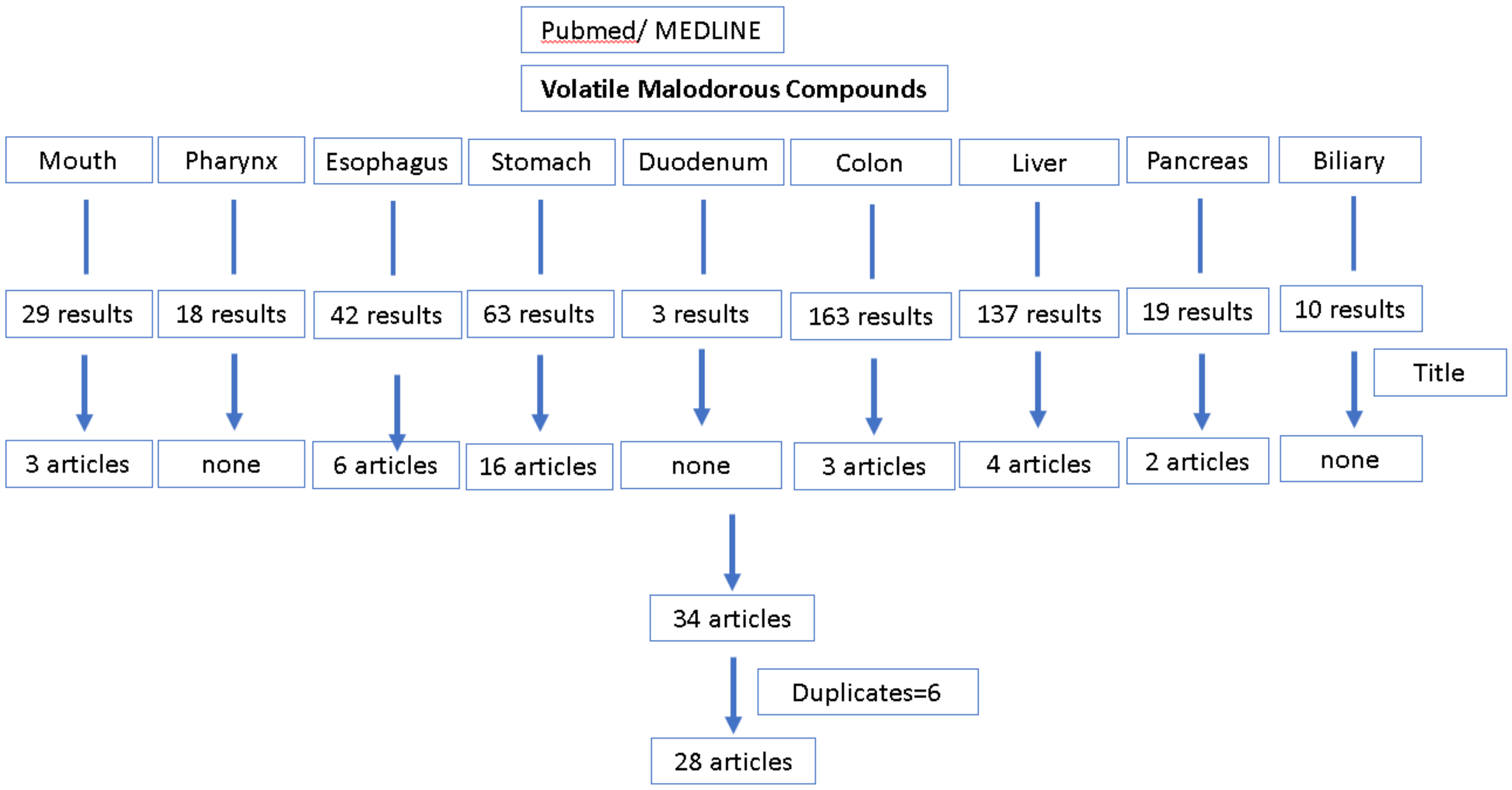

Web of Science

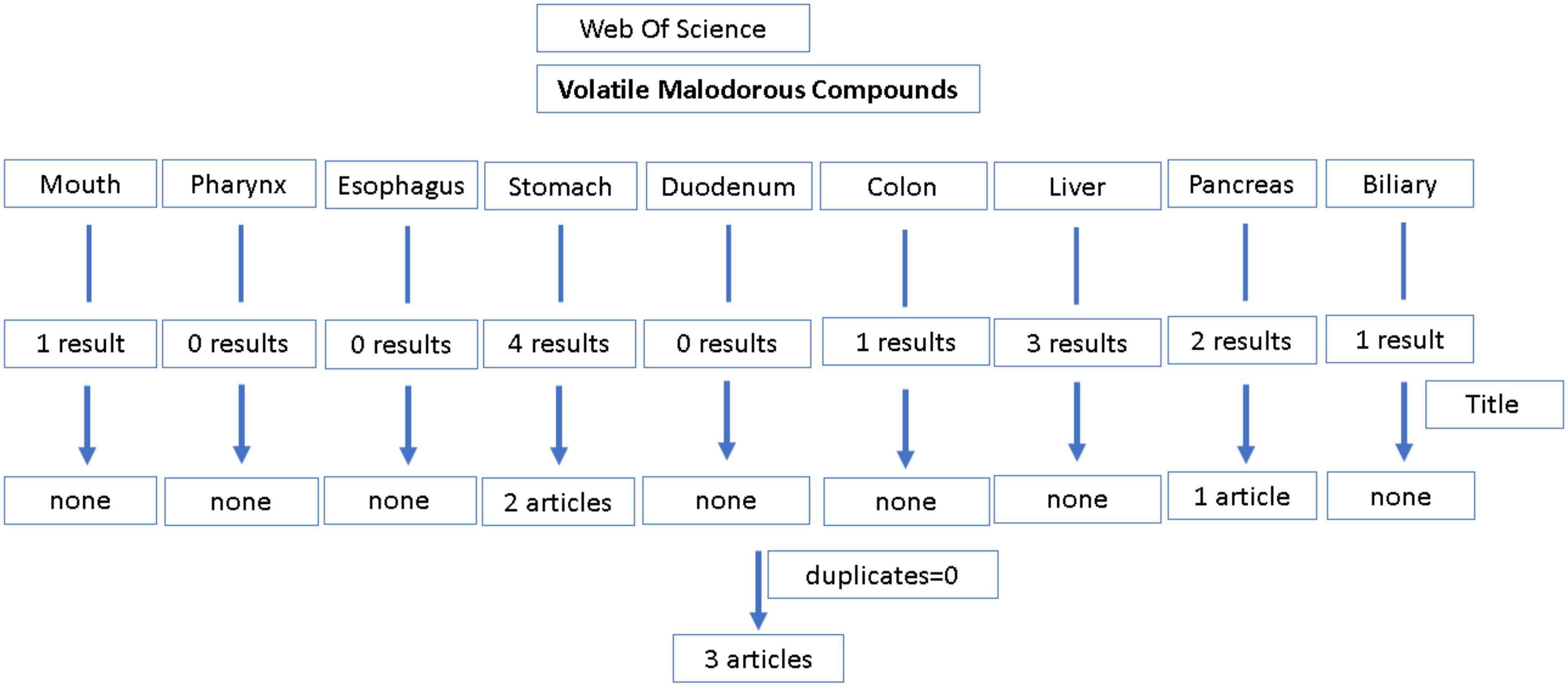

Cochrane Library

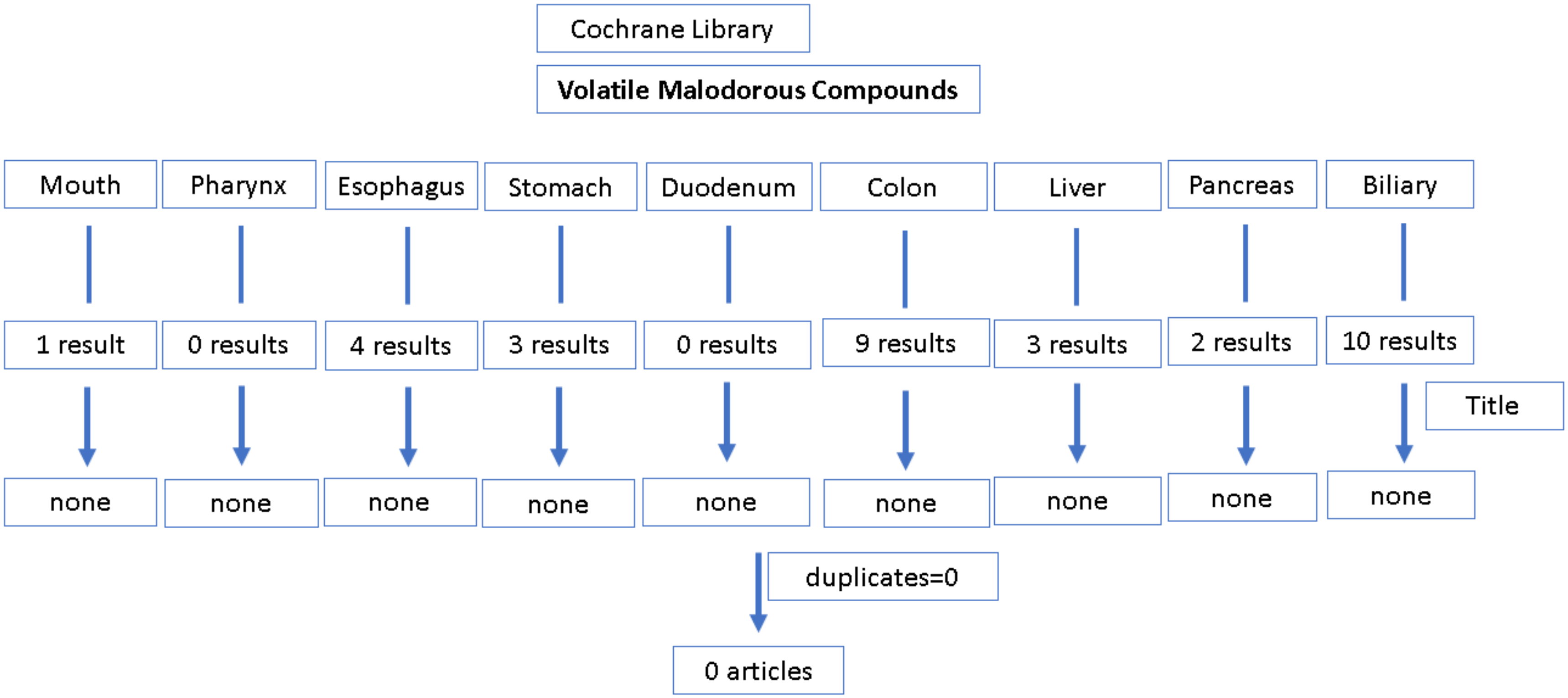

Supplementary File 2. Volatile Organic Compounds in Digestive Neoplasia: 29 Articles identified after the Title and Abstract screening

No	Year	Title / Authors / Journal	Database	
1	2018	Identification of volatile metabolites in human saliva from patients with oral squamous cell carcinoma via zeolite-based thin-film microextraction coupled with GC-MS.<br>Shigeyama H, Wang T, Ichinose M, Ansai T, Lee SW.<br>J Chromatogr B Analyt Technol Biomed Life Sci. 2019 Jan 1;1104:49-58. doi: 10.1016/j.jchromb.2018.11.002. Epub 2018 Nov 6.<br>PMID: 30445287	Pubmed	SALIVA
2	2017	Volatile Organic Compounds in the Breath of Oral Squamous Cell Carcinoma Patients: A Pilot Study.<br>Hartwig S, Raguse JD, Pfitzner D, Preissner R, Paris S, Preissner S.<br>Otolaryngol Head Neck Surg. 2017 Dec;157(6):981-987. doi: 10.1177/0194599817711411. Epub 2017 Jun 6.<br>PMID: 28585450	Pubmed	YES
3	2017	Exhaled breath and oral cavity VOCs as potential biomarkers in oral cancer patients.<br>Bouza M, Gonzalez-Soto J, Pereiro R, de Vicente JC, Sanz-Medel A.<br>J Breath Res. 2017 Mar 1;11(1):016015. doi: 10.1088/1752-7163/aa5e76.<br>PMID: 28165332	Pubmed	YES
4	2019	Mass-Spectrometry Analysis of Mixed-Breath, Isolated-Bronchial-Breath, and Gastric-Endoluminal-Air Volatile Fatty Acids in Esophagogastric Cancer.<br>Adam ME, Fehervari M, Boshier PR, Chin ST, Lin GP, Romano A, Kumar S, Hanna GB.	Pubmed	YES

		Anal Chem. 2019 Mar 5;91(5):3740-3746. doi: 10.1021/acs.analchem.9b00148. Epub 2019 Feb 13.<br>PMID: 30699297		
5	2018	Assessment of a Noninvasive Exhaled Breath Test for the Diagnosis of Oesophagogastric Cancer.<br>Markar SR, Wiggins T, Antonowicz S, Chin ST, Romano A, Nikolic K, Evans B, Cunningham D, Mughal M, Lagergren J, Hanna GB.<br>JAMA Oncol. 2018 Jul 1;4(7):970-976. doi: 10.1001/jamaoncol.2018.0991.<br>PMID: 29799976	Pubmed	YES
6	2018	Cross-platform mass spectrometry annotation in breathomics of oesophageal-gastric cancer.<br>Chin ST, Romano A, Doran SLF, Hanna GB.<br>Sci Rep. 2018 Mar 23;8(1):5139. doi: 10.1038/s41598-018-22890-w.<br>PMID: 29572531	Pubmed	YES
7	2016	Exhaled gases online measurements for esophageal cancer patients and healthy people by proton transfer reaction mass spectrometry.<br>Zou X, Zhou W, Lu Y, Shen C, Hu Z, Wang H, Jiang H, Chu Y.<br>J Gastroenterol Hepatol. 2016 Nov;31(11):1837-1843. doi: 10.1111/jgh.13380.<br>PMID: 26996099	Pubmed	YES
8	2015	Mass Spectrometric Analysis of Exhaled Breath for the Identification of Volatile Organic Compound Biomarkers in Esophageal and Gastric Adenocarcinoma.<br>Kumar S, Huang J, Abbassi-Ghadi N, Mackenzie HA, Veselkov KA, Hoare JM, Lovat LB, Španěl P, Smith D, Hanna GB.<br>Ann Surg. 2015 Dec;262(6):981-90. doi: 10.1097/SLA.0000000000001101.<br>PMID: 25575255	Pubmed	YES

9	2013	Selected ion flow tube mass spectrometry analysis of exhaled breath for volatile organic compound profiling of esophago-gastric cancer.<br>Kumar S, Huang J, Abbassi-Ghadi N, Španěl P, Smith D, Hanna GB.<br>Anal Chem. 2013 Jun 18;85(12):6121-8. doi: 10.1021/ac4010309. Epub 2013 May 29.<br>PMID: 23659180	Pubmed	YES
10	2020	Identification of salivary volatile organic compounds as potential markers of stomach and colorectal cancer: A pilot study.<br>Bel'skaya LV, Sarf EA, Shalygin SP, Postnova TV, Kosenok VK.<br>J Oral Biosci. 2020 Jun;62(2):212-221.	Pubmed	SALIVA
11	2018	Ex vivo emission of volatile organic compounds from gastric cancer and non-cancerous tissue.<br>Mochalski P, Leja M, Gasenko E, Skapars R, Santare D, Sivins A, Aronsson DE, Ager C, Jaeschke C, Shani G, Mitrovics J, Mayhew CA, Haick H.<br>J Breath Res. 2018 Jul 30;12(4):046005. doi: 10.1088/1752-7163/aacbfb.<br>PMID: 29893713	Pubmed	TISSUES
12	2018	Pilot Study: Detection of Gastric Cancer From Exhaled Air Analyzed With an Electronic Nose in Chinese Patients.<br>Schuermans VNE, Li Z, Jongen ACHM, Wu Z, Shi J, Ji J, Bouvy ND.<br>Surg Innov. 2018 Oct;25(5):429-434. doi: 10.1177/1553350618781267. Epub 2018 Jun 18.<br>PMID: 29909757	Pubmed	ELECTR<br>OTHER
13	2017	Volatile organic compounds in gastrointestinal stromal tumour tissue originating from patient-derived xenografts.<br>Wawrzyniak R, Woźniak A, Gebreyohannes YK, Dykcik B, Schöffski P, Markuszewski MJ.	Pubmed<br>Web of<br>Science	TISSUE

		J Breath Res. 2017 Jun 29;11(3):037101. doi: 10.1088/1752-7163/aa6d87.<br>PMID: 28416775		
14	2016	Breath Analysis Based on Surface-Enhanced Raman Scattering Sensors Distinguishes Early and Advanced Gastric Cancer Patients from Healthy Persons.<br>Chen Y, Zhang Y, Pan F, Liu J, Wang K, Zhang C, Cheng S, Lu L, Zhang W, Zhang Z, Zhi X, Zhang Q, Alfranca G, de la Fuente JM, Chen D, Cui D.<br>ACS Nano. 2016 Sep 27;10(9):8169-79. doi: 10.1021/acsnano.6b01441. Epub 2016 Jul 26.<br>PMID: 27409521	Pubmed	YES
15 abstract	2016	Detection of precancerous gastric lesions and gastric cancer through exhaled breath.<br>Amal H, Leja M, Funka K, Skapars R, Sivins A, Ancans G, Liepniece-Karele I, Kikuste I, Lasina I, Haick H.<br>Gut. 2016 Mar;65(3):400-7. doi: 10.1136/gutjnl-2014-308536. Epub 2015 Apr 13.<br>PMID: 25869737	Pubmed	YES
16	2015	Exhaled breath analysis for the diagnosis and assessment of endoluminal gastrointestinal diseases.<br>Markar SR, Wiggins T, Kumar S, Hanna GB.<br>J Clin Gastroenterol. 2015 Jan;49(1):1-8. doi: 10.1097/MCG.0000000000000247.<br>PMID: 25319742	Pubmed	REVIEW
17	2014	Identification of volatile biomarkers of gastric cancer cells and ultrasensitive electrochemical detection based on sensing interface of Au-Ag alloy coated MWCNTs.<br>Zhang Y, Gao G, Liu H, Fu H, Fan J, Wang K, Chen Y, Li B, Zhang C, Zhi X, He L, Cui D.<br>Theranostics. 2014 Jan 5;4(2):154-62. doi: 10.7150/thno.7560. eCollection 2014.<br>PMID: 24465273	Pubmed	OTHER

18	2013	A nanomaterial-based breath test for distinguishing gastric cancer from benign gastric conditions.<br>Xu ZQ, Broza YY, Ionsecu R, Tisch U, Ding L, Liu H, Song Q, Pan YY, Xiong FX, Gu KS, Sun GP, Chen ZD, Leja M, Haick H.<br>Br J Cancer. 2013 Mar 5;108(4):941-50. doi: 10.1038/bjc.2013.44.<br>PMID: 23462808	Pubmed	YES
19	2012	Selected ion flow tube-MS analysis of headspace vapor from gastric content for the diagnosis of gastro-esophageal cancer.<br>Kumar S, Huang J, Cushnir JR, Španěl P, Smith D, Hanna GB.<br>Anal Chem. 2012 Nov 6;84(21):9550-7. doi: 10.1021/ac302409a. Epub 2012 Oct 17.<br>PMID: 23035898	Pubmed	GASTRIC CONTENT
20	2008	Identification of volatile organic compounds secreted from cancer tissues and bacterial cultures.<br>Buszewski B, Ulanowska A, Ligor T, Jackowski M, Kłodzińska E, Szeliga J.<br>J Chromatogr B Analyt Technol Biomed Life Sci. 2008 Jun 1;868(1-2):88-94. doi: 10.1016/j.jchromb.2008.04.038. Epub 2008 May 3.<br>PMID: 18490205	Pubmed	TISSUES
21	2018	Neoplasms and metastasis detection in human blood exhalations with a device composed by nanostructured sensors<br>By: Landini, Nicolo; Anania, Gabriele; Fabbri, Barbara; et al.<br>SENSORS AND ACTUATORS B-CHEMICAL Volume: 271 Pages: 203-214 Published: OCT 15 2018	Web Of Science	OTHER
22	2019	Relationship between cancer tissue derived and exhaled volatile organic compound from colorectal cancer patients. Preliminary results.<br>De Vietro N, Aresta A, Rotelli MT, Zambonin C, Lippolis C, Picciariello A, Altomare DF.	Pubmed	YES

		J Pharm Biomed Anal. 2020 Feb 20;180:113055. doi: 10.1016/j.jpba.2019.113055. Epub 2019 Dec 20.<br>PMID: 31877489		
23	2016	Feasibility of electronic nose technology for discriminating between head and neck, bladder, and colon carcinomas.<br>van de Goor RM, Leunis N, van Hooren MR, Francisca E, Masclee A, Kremer B, Kross KW.<br>Eur Arch Otorhinolaryngol. 2017 Feb;274(2):1053-1060. doi: 10.1007/s00405-016-4320-y. Epub 2016 Oct 11.<br>PMID: 27730323	Pubmed	ELECTR<br>OTHER
24	2010	Detection of lung, breast, colorectal, and prostate cancers from exhaled breath using a single array of nanosensors.<br>Peng G, Hakim M, Broza YY, Billan S, Abdah-Bortnyak R, Kuten A, Tisch U, Haick H.<br>Br J Cancer. 2010 Aug 10;103(4):542-51. doi: 10.1038/sj.bjc.6605810. Epub 2010 Jul 20.<br>PMID: 20648015	Pubmed	YES
25	2018	Profile of exhaled-breath volatile organic compounds to diagnose pancreatic cancer.<br>Markar SR, Brodie B, Chin ST, Romano A, Spalding D, Hanna GB.<br>Br J Surg. 2018 Oct;105(11):1493-1500. doi: 10.1002/bjs.10909. Epub 2018 Jul 18.<br>PMID: 30019405	Pubmed	YES
26	2018	Pancreatic ductal adenocarcinoma can be detected by analysis of volatile organic compounds (VOCs) in alveolar air.<br>Princivalle A, Monasta L, Butturini G, Bassi C, Perbellini L.<br>BMC Cancer. 2018 May 4;18(1):529. doi: 10.1186/s12885-018-4452-0.<br>PMID: 29728093	Pubmed<br>Web Of Science	**YES**
27	2016	Real-time ultrasensitive VUV-PIMS detection of representative endogenous volatile markers in cancers.<br>Li Z, Shu J, Zhang P, Sun W, Yang B, Zhang H.	Pubmed	OTHER

		Cancer Biomark. 2016;16(3):477-87. doi: 10.3233/CBM-160587.<br>PMID: 27062705		
28	2010	The screening of volatile markers for hepatocellular carcinoma.<br>Qin T, Liu H, Song Q, Song G, Wang HZ, Pan YY, Xiong FX, Gu KS, Sun GP, Chen ZD.<br>Cancer Epidemiol Biomarkers Prev. 2010 Sep;19(9):2247-53. doi: 10.1158/1055-9965.EPI-10-0302.<br>PMID: 20826831	Pubmed	**YES**
29	2008	Investigation of volatile biomarkers in liver cancer blood using solid-phase microextraction and gas chromatography/mass spectrometry.<br>Xue R, Dong L, Zhang S, Deng C, Liu T, Wang J, Shen X.<br>Rapid Commun Mass Spectrom. 2008 Apr;22(8):1181-6. doi: 10.1002/rcm.3466.<br>PMID: 18350562	Pubmed	TISSUES

Supplementary File 3. Volatile Organic Compounds in Digestive Neoplasia: Articles Included

No	Title / Authors / Journal	Database	Patients included	Compounds	Methods	Results	Performance's quantification
1	**Volatile Organic Compounds in the Breath of Oral Squamous Cell Carcinoma Patients: A Pilot Study.**<br>Hartwig S, Raguse JD, Pfitzner D, Preissner R, Paris S, Preissner S.<br>Otolaryngol Head Neck Surg. 2017 Dec;157(6):981-987. doi: 10.1177/0194599817711411. Epub 2017 Jun 6.<br>PMID: 28585450	Hartwig S et al., 2017 [1]	Cases:<br>10 patients OSCC before/ after curative surgery for oral cancer<br>(2 tongue, 6 floor of mouth, 1 oropharynx, 1 planum buccal)<br>47-74 years<br>F/M 3/7<br><br>Controls:<br>4 healthy subjects<br>32-48 years<br>F/M 3/1	125 VOCs in patients with OSCC, including:<br><br>alkanes, alkenes, aromatic and chlorinates hydrocarbons, terpenes, siloxanes, monovalent alcohols, esters, ketones, cyclic ethers, glycols, higher aldehydes, fatty acids esters, heterocycles, phenols, microbial VOCs.	Breath samples collected in Tenax tubes<br>Capillary gas chromatography coupled with a Mass spectrometer	Signature of 8 compounds for oral cancer:<br>DBH,dibutylhydroxytoluene; DDS, dimethyl disulfide;D5,decamethylcyclopentasiloxane; MEK, methyl ethyl ketone; NHE, n-heptane; PX, p-xylene; TOL, toluene; 1-HE, 1-heptene<br>3 compounds absent after cancer surgery:<br>- Dimethyl disulfide (DDS) decreased 4 /10, absent 5/10<br>-Decamethylcyclopenta-siloxane (D5) absent 5/10<br>- P-xylene (PX) absent 5/10<br>5 compounds increased after cancer surgery:<br>- Methyl ethyl ketone (MEK)<br>- Dibutylhydroxytoluene (DBH)<br>- N-heptane (NHE)<br>- toluene (TOL)<br>- 1-heptene (1H)	--
2	**Exhaled breath and oral cavity VOCs as potential biomarkers in oral cancer patients.**<br>Bouza M, Gonzalez-Soto J, Pereiro R, de Vicente JC, Sanz-Medel A.	Bouza M et al., 2017 [2]	Cases:<br>26 OSCC patients<br>36-84 years<br>F/M 2/2	105 VOCs identified:<br><br>undecane,<br>dodecane,<br>decanal,	Exhaled breath samples collected<br>Analysis by solid-phase microextraction	Correlated with:<br>Tumor size<br>- Benzaldehyde<br>- 3,7-dimethylundecane	--

	J Breath Res. 2017 Mar 1;11(1):016015. doi: 10.1088/1752-7163/aa5e76.<br>PMID: 28165332		Controls:<br>26 cancer-free controls<br>21-69 years<br>F/M 4/10	benzaldehyde,<br>3,7-dimethyl undecane,<br>4,5-dimethyl nonane,<br>1-octene, and<br>Hexadecane<br><br>Potential biomarkers in oral cavity:<br>toluene<br>styrene<br>benzaldehyde<br>nonanal<br>decanal<br>benzyl alcohol<br>dodecanal<br>2-ethyl-1-hexanol	Gas chromatography—mass spectrometry detection	Histological degree of differentiation<br>- Benzaldehyde<br>- Butyl acetate<br><br>Tumor recurrence<br>- Benzaldehyde	
3	**Mass-Spectrometry Analysis of Mixed-Breath, Isolated-Bronchial-Breath, and Gastric-Endoluminal-Air Volatile Fatty Acids in Esophagogastric Cancer**.<br>Adam ME, Fehervari M, Boshier PR, Chin ST, Lin GP, Romano A, Kumar S, Hanna GB.<br>Anal Chem. 2019 Mar 5;91(5):3740-3746. doi: 10.1021/acs.analchem.9b00148. Epub 2019 Feb 13.<br>PMID: 30699297	Adams ME et al., 2019 [3]	Cases:<br>25 patients with esophagogastric cancer<br><br>Controls:<br>20 control subjects	Volatile fatty acids (VFAs):<br><br>acetic acid<br>butyric acid<br>pentanoic acid<br>hexanoic acid	Samples:<br>- ex vivo headspace above underivatized tissues<br>- in vivo analysis within defined anatomical compartments:<br>- mixed breath,<br>- isolated bronchial breath<br>- gastric-endoluminal air	Significant increased levels of VFAs in esophagogastric cancer/ headspace concentrations:<br>- acetone<br>- acetic acid<br>- butyric acid<br>- pentanoic acid<br>- hexanoic acid<br>VFAs increased in mixed breath in esophagogastric cancer:<br>- acetic acid<br>- butyric acid<br>- pentanoic acid	--
4	**Assessment of a Noninvasive Exhaled Breath Test for the Diagnosis of Oesophagogastric Cancer.**<br>Markar SR, Wiggins T, Antonowicz S, Chin ST, Romano A, Nikolic K, Evans B,	Markar SR et al., 2018 [4]	Cases:<br>163 OGC patients<br>68 (60-75) years<br>F/M 91/81<br><br>Controls:	5-VOCs model:<br>butyric acid,<br>pentanoic acid,<br>hexanoic acid,<br>butanal,<br>decanal.	Ion flow tube mass spectrometry (SIFT-MS) analysis.<br>Gas chromatography	Significant differences between cancer and control group:<br><br>- butyric acid,<br>- hexanoic acid,	Breath test to diagnose esophagogastr ic cancer – ROC:<br>Sensitivity 80%

	Cunningham D, Mughal M, Lagergren J, Hanna GB.<br>JAMA Oncol. 2018 Jul 1;4(7):970-976. doi: 10.1001/jamaoncol.2018.0991.<br>PMID: 29799976		172 control group<br>55 (41-75) years<br>F/M 91/81		mass spectrometry (GC-MS).	- butanal,<br>- decanal.	Specificity 81%.
5	**Cross-platform mass spectrometry annotation in breathomics of oesophageal-gastric cancer.**<br>Chin ST, Romano A, Doran SLF, Hanna GB.<br>Sci Rep. 2018 Mar 23;8(1):5139. doi: 10.1038/s41598-018-22890-w.<br>PMID: 29572531	Chin ST et al., 2018 [5]	Oesophageal-gastric cancer<br>21 cases	24 VOCs tested, most frequent:<br>- Acetone<br>- Phenol<br>- Benzaldehyde<br>- Butanal<br>- Nonanal<br>- Decanal	Exhaled breath sampled onto thermal desorption unit (TD) tubes<br>Mass spectrometry analysis	Positive chemical ionization – Mass spectrometry (PCI-MS) analysis: molecular ion and fragments i.e. [M + H]+, followed by [M-17]+ and [M-73]+ could be potentially used as the diagnostic ions to distinguish the corresponding VOCs	--
6	**Exhaled gases online measurements for esophageal cancer patients and healthy people by proton transfer reaction mass spectrometry.**<br>Zou X, Zhou W, Lu Y, Shen C, Hu Z, Wang H, Jiang H, Chu Y.<br>J Gastroenterol Hepatol. 2016 Nov;31(11):1837-1843. doi: 10.1111/jgh.13380.<br>PMID: 26996099	Zou X et al., 2016 [6]	Cases:<br>29 patients with esophageal cancer<br>61.3±9.4years<br>M/F 27/2<br><br>Controls:<br>57 healthy people<br>55.4±8.6<br>M/F 28/29	7 ions, of which:<br><br>m/z63=dimethyl sulfide (830.9), thioethanol (789.6)<br><br>m/z95=phenol (817.3), 1,3-cycloheptadiene (776.5); dimethylsulfone (819.4); 2-methyl-1,3-diazine (917.4); 1-methylene-2-cyclohexene (893.5)<br><br>m/z107=ethylbenzene (788.0), p-xylene (794.4), o-xylene (796.0), m-xylene (812.1), benzaldehyde (834.0)	Flow tube mass spectrometry (SIFT-MS) and<br>Proton transfer reaction mass spectrometry (PTR-MS)<br><br>Hand-made	5 ions decreased:<br>- m/z 34,<br>- m/z 63<br>Dimethyl sulfide<br>Thioethanol,<br>- m/z 95<br>Phenol<br>1, 3-cycloheptadiene,<br>- m/z 107<br>Ethylbenzene<br>P-xylene<br>O-xylene<br>M-xylene<br>Benzaldehyde,<br>- m/z 45<br>Acetaldehyde<br>Ethylene oxide.<br>2 ions increased:<br>- m/z 136,<br>- m/z 27.	Sensitivity 86.2%<br>Specificity 89.5%<br>AUC 0.943<br>Discriminant accuracy<br>Stage 1 100%<br>Stage 2 71%<br>Stage 3 86%<br>Stage 4 93%

				m/z45 = acetaldehyde (768.5), ethylene oxide (774.2)			
7	**Selected ion flow tube mass spectrometry analysis of exhaled breath for volatile organic compound profiling of esophago-gastric cancer.**<br>Kumar S, Huang J, Abbassi-Ghadi N, Španěl P, Smith D, Hanna GB.<br>Anal Chem. 2013 Jun 18;85(12):6121-8. doi: 10.1021/ac4010309. Epub 2013 May 29.<br>PMID: 23659180	Kumar S et al., 2013 [8]	Three groups:<br>- with gastro-esophageal cancer,<br>- noncancer diseases of the upper gastro-intestinal tract,<br>- healthy controls	17 VOCs investigated:<br>methanol<br>propanol<br>butanol<br>pentanol<br>pentanoic acid<br>hexanoic acid<br>hydrogen sulphide<br>hydrogen cyanide<br>acetaldehyde<br>formaldehyde<br>acetone<br>acetic acid<br>isoprene<br>phenol<br>methyl phenol<br>ethyl phenol<br>ammonia	Exhaled breath analysis by the multiple ion monitoring (MIM) mode of the selected ion flow tube mass spectrometry (SIFT-MS)	Significant difference cancer/ control for 4 VOCs:<br><br>- hexanoic acid,<br>- phenol,<br>- methyl phenol,<br>- ethyl phenol	ROC curve: esophago-gastric cancer from positive controls AUC = 0.91.
8	**Mass Spectrometric Analysis of Exhaled Breath for the Identification of Volatile Organic Compound Biomarkers in Esophageal and Gastric Adenocarcinoma.**<br>Kumar S, Huang J, Abbassi-Ghadi N, Mackenzie HA, Veselkov KA, Hoare JM, Lovat LB, Španěl P, Smith D, Hanna GB.<br>Ann Surg. 2015 Dec;262(6):981-90. doi: 10.1097/SLA.0000000000001101.<br>PMID: 25575255	Kumar S et al., 2015 [7]	Cases:<br>48 patients esophageal cancer 63.5 (55.3–72.8) years<br>M/F 40/8<br><br>33 patients gastric adenocarcinoma 58 (50.5–70.5) years<br>M/F 24/9<br><br>Controls:<br>16 patients Barrett metaplasia	29 VOCs from 8 major chemical groups:<br><br>alcohols,<br>phenols,<br>ketones,<br>fatty acids,<br>aldehydes,<br>sulfur-containing compounds,<br>nitrogen compounds,<br>ether.	Selected Ion Flow Tube Mass Spectrometry (SIFT-MS) – Profile 3<br><br>Multiple Ion Monitoring Mode<br><br>Instrument Science Crewe, United Kingdom	Significantly increased in both cancers:<br><br>- Pentanoic acid<br>- Hexanoic acid<br>- Phenol<br>- Methyl phenol<br>- Ethyl phenol<br>- Butanal<br>- Pentanal<br>- Hexanal<br>- Heptanal<br>- Octanal<br>- Nonanal<br>- Decanal	Esophageal cancer vs Normal<br>AUC 0.97<br>Sensitivity 98%<br>Specificity 91.7%<br><br>Gastric adenocarcinoma vs Normal<br>AUC 0.98<br>Sensitivity 100%<br>Specificity 92.2%

			67 (58.8–72.8) years<br>M/F 11/5<br><br>62 patients benign upper gastrointestinal disease<br>64.5 (50–72) years<br>M/F 38/24<br><br>51 patients with normal upper gastrointestinal tract<br>61 (50–73) years<br>M/F 30/21			Significantly increased in esophageal cancer:<br>- Butyric acid<br><br>No difference:<br>- Methanol<br>- Acetone<br>- Ammonia Isoprene	
9	**Breath Analysis Based on Surface-Enhanced Raman Scattering Sensors Distinguishes Early and Advanced Gastric Cancer Patients from Healthy Persons.**<br>Chen Y, Zhang Y, Pan F, Liu J, Wang K, Zhang C, Cheng S, Lu L, Zhang W, Zhang Z, Zhi X, Zhang Q, Alfranca G, de la Fuente JM, Chen D, Cui D.<br>ACS Nano. 2016 Sep 27;10(9):8169-79. doi: 10.1021/acsnano.6b01441. Epub 2016 Jul 26.<br>PMID: 27409521	Chen Y et al., 2016 [9]	Three groups:<br>- early gastric cancer (EGC)<br>- advanced gastric cancer (AGC)<br>- healthy persons	14 VOCs analyzed:<br>isoprene<br>menthol<br>pivalic acid<br>acetone<br>tetradecane<br>ethyl-pentane<br>3-methylpentane<br>hexane<br>hexanol<br>2,3-dimethylpentane<br>2-methylpentane<br>2-methylhexane<br>dodecane<br>phenyl acetate	Gas chromatography–mass spectrometry coupled with solid phase microextraction (SPME)	> 80% of healthy, but barely existed in EGC breath samples:<br>- Isoprene (peak 3)<br>- menthol (peak 12)<br><br>> 80% of GC breath:<br>- the pivalic acid (peak 9)<br><br>> 70% of EGC more than those in the AGC samples:<br>- acetone (peak 1)<br>- tetradecane (peak 13)<br><br>> 65% AGC samples:<br>- 2-Methylpentane (peak 2)<br>- 3-methylpentane (peak 4)<br>- hexane (peak5)	Sensitivity 83%<br>Specificity 92%

						- 2,3-dimethylpentane (peak 6)<br>- 2-methylhexane (peak 7)<br>- 2-methylhexane (peak 8)<br>- dodecane (peak 14)	
10	**A nanomaterial-based breath test for distinguishing gastric cancer from benign gastric conditions.**<br>Xu ZQ, Broza YY, Ionsecu R, Tisch U, Ding L, Liu H, Song Q, Pan YY, Xiong FX, Gu KS, Sun GP, Chen ZD, Leja M, Haick H.<br>Br J Cancer. 2013 Mar 5;108(4):941-50. doi: 10.1038/bjc.2013.44.<br>PMID: 23462808	Xu ZQ et al., 2013 [10]	130 patients with gastric complaints:<br>- 37 gastric cancer<br>- 32 gastric ulcer<br>- 61 less severe conditions<br><br>17 patients - Early stage (stages I and II)<br>57.6±11.7years<br>F/M 4/13<br><br>18 patients - Late stage (stages III and IV)<br>59.1±6.9<br>F/M 5/13<br><br>2 patients unknown stage<br><br>32 patients gastric ulcer<br>50.8±14.2 years<br>F/M 9/23<br><br>29 patients - endoscopic abnormalities without ulceration	214 VOCs in more than 85% of the breath samples<br><br>prominent peaks corresponding to five VOCs	Exhaled alveolar breath collected<br>Gas-chromatography/mass spectrometry (GC-MS)	Eight !!!! five VOCs increased in gastric cancer or ulcer versus less severe conditions:<br>- 2-propenenitrile<br>- 2-butoxy-ethanol<br>- furfural<br>- 6-methyl-5-hepten-2-one<br>- isoprene	Three discriminant factor analysis (DFA) pattern developed:<br><br>- GC vs benign gastric condition sensitivity 89%; specificity 90%<br><br>- early stage gastric cancer (I and II) vs late stage (III and IV), for gastric cancer sensitivity 89% specificity 94%<br><br>- ulcer vs less severe, among benign conditions sensitivity 84% specificity 87%

			50.6±9.3 years<br>F/M 12/15<br><br>No endoscopic abnormalities<br>52.2±8.3 years<br>F/M 19/13				
11	**Detection of precancerous gastric lesions and gastric cancer through exhaled breath.**<br>Amal H, Leja M, Funka K, Skapars R, Sivins A, Ancans G, Liepniece-Karele I, Kikuste I, Lasina I, Haick H.<br>Gut. 2016 Mar;65(3):400-7. doi: 10.1136/gutjnl-2014-308536. Epub 2015 Apr 13.<br>PMID: 25869737	Amal H et al., 2015 [11]	total 484 patients:<br><br>- 99 patients with gastric cancer (GC): 36 patients non-advanced (I-II) GC 59 patients advanced (III-IV) GC<br>4 patients un-staged GC<br><br>OLGIM - operative link on gastric intestinal metaplasia<br><br>- 155 patients OLGIM 0<br>- 136 patients OLGIM I-II<br>- 34 patients OLGIM III-IV<br><br>- 53 patients with peptic ulcer disease (PUD)<br><br>- 7 patients with dysplasia	130 VOCs originally obtained from the exhaled breath samples in various study groups	First analysis: gas chromatography linked to mass spectrometry (GCMS)<br><br>Second analysis: cross-reactive nanoarrays combined with pattern recognition	8 VOCs with significant difference between groups:<br><br>- 2-propenenitrile<br>- furfural<br>- 2- butoxy-ethanol<br>- hexadecane<br>- 4-methyloctane<br>- 1,2,3-tri-methylbenzene<br>- α-methyl-styrene<br>- 2-butanone	nanoarray analysis GC and the control group (OLGIM 0–IV) sensitivity 73%, specificity 98%, accuracy 92%<br><br>Between groups:<br>GC versus OLGIM 0–II: 97%, 84% and 87%;<br>GC versus OLGIM III–IV: 93%, 80% and 90%<br>OLGIM I–II versus OLGIM III– IV and dysplasia combined: 83%, 60% and 61%<br><br>OLGIM - operative link

							on gastric intestinal metaplasia
12	**Relationship between cancer tissue derived and exhaled volatile organic compound from colorectal cancer patients. Preliminary results.**<br>De Vietro N, Aresta A, Rotelli MT, Zambonin C, Lippolis C, Picciariello A, Altomare DF.<br>J Pharm Biomed Anal. 2020 Feb 20;180:113055. doi: 10.1016/j.jpba.2019.113055. Epub 2019 Dec 20.<br>PMID: 31877489	De Vietro N et al., 2020 [12]	colorectal cancer (CRC)<br><br>VOCs exhaled by 7 CRC patients<br>65 ± 10years<br>F/M 3/4<br><br>Compared to:<br>cancer tissue<br>normal colonic mucosa<br>60 ± 7 years<br>F/M 12/8	At least 4 CRC:<br><br>acetone<br>acetonitrile<br>benzaldehyde<br>benzene, ethyl<br>benzene, methyl<br>butanoic acid<br>decanal<br>diallyl disulfide dodecane<br>dodecanoic acid<br>eptanal<br>hexanal<br>indole<br>nonanal<br>octanoic acid<br>pentanal<br>pentanoic acid<br>phenol<br>propanoic acid<br>tetradecane	Gas Chromatography with Mass Spectrometry (GC–MS)	VOCs most frequently detected both in the exhaled breath and secreted by tissues also:<br><br>- benzaldehyde<br>- benzene ethyl<br>- benzene methyl<br>- butanoic acid<br>- dodecanoic acid<br>- indole<br>- nonanal<br>- octanoic acid<br>- pentanoic acid<br>- phenol<br>- tetradecane<br><br>In particular, significantly different in cancer tissue versus normal colonic mucosa:<br>- benzaldehyde<br>- benzene ethyl<br>- indole	--
13	**Detection of lung, breast, colorectal, and prostate cancers from exhaled breath using a single array of nanosensors.**<br>Peng G, Hakim M, Broza YY, Billan S, Abdah-Bortnyak R, Kuten A, Tisch U, Haick H.<br>Br J Cancer. 2010 Aug 10;103(4):542-51. doi: 10.1038/sj.bjc.6605810. Epub 2010 Jul 20.<br>PMID: 20648015	Peng G et al., 2010 [13]	177 volunteers<br>20-75 years<br><br>96 volunteers<br>30–75 years<br>30 primary lung cancer patients<br>26 primary colon cancer patients<br>22 primary breast cancer patients	16 VOCs selected:<br><br>1-methyl-4-(1-methylethyl)benzene<br>toluene<br>dodecane<br>3,3-dimethyl pentane<br>2,3,4-trimethyl hexane<br>1,10-(1-butenylidene)bis benzene<br>1,3-dimethyl benzene	Exhaled alveolar breath<br>Gas chromatography linked to the mass spectrometry technique (GC-MS)<br>Solid phase microextraction (SPME)	2,6,11-trimethyl-dodecane: 80% males, but in none of the females<br><br>3,7-dimethyl-undecane in 100% of the subjects with allergies, but only in 9% of the subjects without allergies	

			18 primary prostate cancer patients<br>22 healthy controls	1-iodo nonane<br>(1,1-dimethylethyl)thio acetic acid<br>4-propylcyclohexyl)-40-cyano[1,10-biphenyl]-4-yl ester benzoic acid<br>2-amino-5-isopropyl-8-methyl-1-azulenecarbonitrile 5-(2-methylpropyl)nonane<br>2,3,4-trimethyl decane<br>6-ethyl-3-octyl ester 2-trifluoromethyl benzoic acid xylene<br>2,2-dimethyl decane<br><br>6 VOCs tested for colon cancer:<br>- 1,10-(1-butenylidene)bis benzene<br>- 1,3-dimethyl benzene<br>- 1-iodo nonane<br>- 1,1-dimethylethyl)thio acetic acid<br>- 4-(4-propylcyclohexyl)-40-cyano[1,10-biphenyl]-4-yl ester benzoic acid<br>- 2-amino-5-isopropyl-8-methyl-1-azulenecarbonitrile		Low sensitivity, 30%, for colon cancer	
14	**Profile of exhaled-breath volatile organic compounds to diagnose pancreatic cancer.**<br>Markar SR, Brodie B, Chin ST, Romano A, Spalding D, Hanna GB.	Markar SR et al.,2018 [14]	68 patients for the development cohort:<br>- 25 with cancer<br>70 (62–77) years<br>F/M 10/15<br>- 43 no cancer	66 VOCs identified, 22 excluded (not endogeneous):<br>44 VOCs analyzed	Breath collected using in steel breath bags.<br>Mass spectrometric analysis: TD–gas chromatography	12 VOCs were significantly different between groups.<br><br>raised in cancer:<br>- formaldehyde<br>- acetone	cancer from no cancer:<br>AUC=0.736<br>sensitivity 81%<br>specificity 58%

	Br J Surg. 2018 Oct;105(11):1493-1500. doi: 10.1002/bjs.10909. Epub 2018 Jul 18.<br>PMID: 30019405		60 (44–72) years<br>F/M 22/21<br><br>64 patients for the validation cohort:<br>- 32 with cancer<br>68 (61–72) years<br>F/M 11/21<br>- 32 no cancer<br>58 (49–74)<br>F/M 11/21		(GC)–mass spectrometry (MS)	- acetoin<br>- undecane<br>- isopropyl alcohol<br><br>decreased in cancer:<br>- pentane<br>- n-hexane<br>- 1-butanol<br>- 1-(methylthio)-propane<br>- benzaldehyde<br>- tetradecane<br>- amylene hydrate	adenocarcinoma from no cancer:<br>AUC=0.744<br>sensitivity 70%<br>specificity 74%
15	**Pancreatic ductal adenocarcinoma can be detected by analysis of volatile organic compounds (VOCs) in alveolar air.**<br>Princivalle A, Monasta L, Butturini G, Bassi C, Perbellini L.<br>BMC Cancer. 2018 May 4;18(1):529. doi: 10.1186/s12885-018-4452-0.<br>PMID: 29728093	Princivalle A et al., 2018 [15]	- 219 subjects<br>- 52 (21-83) years<br><br>144 controls<br>47 (21-78) years<br><br>75 patients pancreatic ductal adenocarcinoma (PDA)<br>62 (23-83) years<br><br>Initial tests:<br>65 cases<br>102 controls<br><br>Validation:<br>23 cases<br>26 controls	92 compounds	end-tidal breath: one deep exhalation inside a hand-device ion-molecule reaction mass spectrometry	final predictive model: 10 VOCs<br><br>Increased in PDA:<br>- M17 ammonia,<br>- M43 acetyl group,<br>- M71,<br>- M74,<br>- M89,<br>- M112.<br><br>Decreased in PDA:<br>- M34 hydrogen sulfide,<br>- M44 acetaldehyde,<br>- M62,<br>- M64 sulfur dioxide .	sensitivity 100%<br>specificity 84%<br>ROC curve AUC=0.99
16	**The screening of volatile markers for hepatocellular carcinoma.**<br>Qin T, Liu H, Song Q, Song G, Wang HZ, Pan YY, Xiong FX, Gu KS, Sun GP, Chen ZD.	Qin T et al., 2010 [16]	Three groups:<br><br>- 30 hepatocellular carcinoma (HCC) patients comorbid	3-hydroxy-2-butanone<br>styrene<br>decane	gas chromatography mass spectrometry	best diagnostic value:<br>- 3-hydroxy-2-butanone	HCC patients versus normal control:<br>sensitivity 86.7%

	Cancer Epidemiol Biomarkers Prev. 2010 Sep;19(9):2247-53. doi: 10.1158/1055-9965.EPI-10-0302. PMID: 20826831		with type B hepatitis and cirrhosis<br>- 27 hepatocirrhosis patients<br>- 36 healthy volunteers				specificity 91.7%<br><br>cross-validation: sensitivity 83.3% specificity 91.7%

Abbreviations*:* GC-MS - Gas chromatography mass spectrometry; OLGIM - operative link on gastric intestinal metaplasia; OSCC - oral squamous cell carcinoma; OGC - esophagogastric cancer; MIM - multiple ion monitoring; PUD - peptic ulcer disease ; VFAs - volatile fatty acids; VOCs - Volatile Organic Compounds; SIFT-MS - selected ion flow tube mass spectrometry; SPME - Gas chromatography– mass spectrometry coupled with solid phase microextraction.
